# Experimental models of chemical carcinogenesis: bridging toxicology and preclinical pharmacology

**DOI:** 10.1186/s43046-026-00345-y

**Published:** 2026-02-23

**Authors:** Tejaswi, Rahul Singh, Poonam Yadav, Mohit Kumar, Anshu Sharma

**Affiliations:** School of Healthcare & Allied Sciences, GD Goenka University, 122018 Sohna, India

**Keywords:** Cancer, Carcinogens, Experimental, Therapeutic, Tumor

## Abstract

Those experimental animals that tend to develop primary malignancies as a result of direct exposures to synthetic cancer agents can very well represent early stages of human cancers as compared to transplanted as well as genetically engineered tumor models. Generally, malignant growth resulting due to cancer agents tends to undergo three phases: initiation, promotion, as well as progression. On the basis of genetic alteration, agents can be divided into genotoxgents as well as non-genotoxgents. Carcinogens that tend to be complete, incomplete, as well as specified towards a target organ are classified into the other categories. These agents can be found through environmental pollutants, meat products, food additives, as well as anticancer agents.

The advantages associated with the primary models created in animals by using chemicals include relevance to human clinical models, ease of production, and efficiency. However, it is difficult to track these tumors in small animals without damaging their bodies. As in human models, they grow randomly concerning site, timing, and number in animal models too.

Non-invasive application of magnetic resonance imaging techniques has however shown promise in the diagnosis and follow-up of these models of cancer. This is because there are a number of advantages associated with the use of the MRI technology, among them being that this technology does not carry the hazards of ionizing radiation.

This review evaluates the unique translational utility of chemically induced primary cancer models, which, unlike traditional xenografts, allow for the study of the entire carcinogenic spectrum—from initiation to progression. The aim of this work is to highlight the novelty of these models in bridging the gap between toxicology and clinical pharmacology, specifically their role in validating non-invasive imaging biomarkers and screening chemopreventive agents—such as Rapamycin and Resveratrol—currently under clinical investigation. By integrating these models with advanced MRI techniques, researchers can achieve more accurate preclinical staging and treatment monitoring. Ultimately, we argue that these models are essential for the development of targeted therapies and diagnostic agents that more faithfully replicate the sporadic nature of human clinical oncology.

## Introduction

Cancer is a complex disease which often begins with local symptoms and progresses through a multistep process defined by several key characteristics. These hallmarks include rapid growth, resistance to cell death, the formation of new blood vessels (neoangiogenesis), local tissue invasion, and the spread of cancer to distant sites (metastasis). Due to the diversity of pathological features, genetic variations, and patient outcomes in clinical oncology, it is challenging to identify a single animal model that accurately replicates this complex process.

A fundamental classification of these agents is based on their interaction with genetic material. Genotoxic carcinogens are primary initiators that directly damage DNA, often leading to irreversible mutations. In contrast, non-genotoxic carcinogens (NGCs) do not interact with DNA directly but drive tumorigenesis through mechanisms such as chronic inflammation, hormonal imbalance, or epigenetic modifications. Distinguishing between these mechanisms is vital for accurate risk assessment and the development of targeted prevention strategies [1]. 

Consequently, various animal cancer models have been developed, including genetically engineered models, transplanted tumors and environmentally induced cancer, such as those caused by chemical exposure. These models are designed to mimic non-identical human cancer pathology and to address a wide range of research questions.

Transplantable tumor models involve transferring cancer cells or tissue from one animal (the donor) to another (the recipient). Such models are considered to be either xenograft or allograft models established on the species of the organisms being used and the genetic background of the organisms being studied. In xenograft models, the host animals are typically immunocompromised, allowing them to accept cancer cells from other species more easily. By contrast, allografts refer to transplants between members of the same species. The transplantation may be done either through orthotopic implantation (performing the implantation in the original location of the tumor), subcutaneous, or intravascular implantation (implantation through the bloodstream) to investigate different phases of the disease [1–3]. 

Genetically modified models (GEMs) can be generated by modifying the genes found in an organism by either overexpressing an oncogene or by introducing a mutation to the tumor suppressor gene by making it inactive. GEMs consist of two types: Transgenic models/endogenous models.

Researchers are able to investigate the role of particular genes for the development of tumors using genetically modified models. Although the generation of genetically modified mouse models is rather complex, most genetically modified mice models accurately resemble human cancers [1]. 

Genetically Modified Models (GEMs) are useful in the study of inherited cancers, but approximately 90% of cancers in human beings are caused by environmental/chemical exposures. Chemically induced models are very important since they replicate the cause in the form of an environment and provide an opportunity to investigate the “promotion” phase, since it is entirely absent in Xenograft models but is very important in identifying methods to halt cancer development. More often than not, researchers are utilizing these animal studies and bioinformatics tools combined with AI in order to compare their outputs with human studies [2]. 

These models mimic the complete range of cancer evolution and advancement, from initial onset to clinical emergence and later phases. They are essential for exploring the causes, prevention, diagnosis, and treatment of cancer all over the stages. The main tumor lesions identified in these models frequently display histological complexity and diversity akin to that of human cancers.

Chemically induced models for cancer induction are especially beneficial because of their non-invasive or minimally invasive characteristics, high tumor load, capacity to mimic various cancer types, and reproducibility. This model accurately replicates the development of human cancer from the beginning stage, serving as an essential resource for cancer research [4, 5].

By mimicking the complete range of cancer evolution, these models provide a unique window into the presence of malignancy during the initial stages is of utmost significance. This helps in making sure that there is the presence of elementary indications of malignancy within the early stages of cancers, which can either be genetic signs or signs of precancerous lesions. The aforementioned step holds the highest level of importance that can assist in deciding the presence of cancers within their initial stages by using non-invasive techniques [6, 7]. 

## Primary cancer development mechanisms

The basic process of how cancers occur is a very complex process that involves multiple pathways. There is chemical carcinogenesis, which occurs throughout the three stages of initiation, promotion, and progression. The fundamental biological process that takes place during the cellular process of the development of cancers involves the interfacing with the DNA, RNA, and proteins present in the cells, which may contain genetic mutations [8–11].

### Tumor initiation, promotion, and progression

Cancer stands as a complicated disease, genetically fueled, that results in the uncontrolled growth and development of cells due to the uncontrolled mutation of genes within them. The pictorial representation models the process of chemical carcinogenesis, initiation, promotion, and progression, all of which have a pivotal position within the multi-factor process of cancer development. These processes do not take place as individual occurrences but as a complex interaction between them that takes normal cells towards malignancy [8–11].

In the initiation phase, the cellular environment is first exposed to initiators, typically chemical agents that undergo metabolic activation by enzymes within the body. Once transformed, these initiators can bind covalently to DNA, resulting in permanent genetic mutations. This graphic aptly conveys how one cell posits the importance of genetic change, frequently by the binding of the initiator to DNA through a covalent bond, to be “initiated.” Notably, this is an irreversible and heritable process. This means that all daughter cells arising from the cell will possess this change and are ready to be transformed when the time is right [9].

The next phase after initiation is the promotion phase, which explains the action of external factors named promoters on the mutated cells. As distinct from initiators, promoters do not induce DNA damage or enter into covalent bindings with large molecules directly. Rather, they bond with cell receptors, evoking cell growth as their target responds favorably to their action due to the enhanced clonal expansion of mutated cells, as depicted below: This phase has the characteristic of being reversible and contingent on constant exposure to the promoter, offering an avenue for possible interference [10].

The Progression Phase i.e. the stage where the expanded cells change to become of a cancerous nature. Other mutations, as well as chromosomal aberrations such as aneuploidy, will occur, resulting in an unstable genome. The stage will be characterized by heightened cell proliferation, invasion, and ultimately metastasis. The stage shall be represented by the cell picture, where a mass of different cell morphology shall be depicted, symbolizing the stage of malignancy. The stage shall denote the onset of cancer, which will be relatively more aggressive with an ominous prognosis [11]. 

Altogether, this sequence—from the initial DNA lesion to tumor formation—underscores the importance of understanding each phase within the broader context of cancer prevention and treatment. Targeting the early stages, particularly initiation and promotion, presents promising opportunities for intervention, while insights into the progression phase can inform therapeutic strategies aimed at halting or reversing tumor development [1]. 

Their dose-response relationships characterize the distinction between genotoxic and non-genotoxic pathways. Genotoxic agents are generally considered to have no safe ‘threshold,’ as even low-level exposure can trigger a mutation. Conversely, non-genotoxic carcinogens exhibit a clear threshold effect; they require sustained exposure at specific levels to alter cellular environments or activate signaling pathways. Recent research highlights that NGCs specifically target non-coding RNAs and epigenetic ‘readers’ to bypass direct DNA damage while still achieving malignant transformation [6]. 

### Carcinogens, both genotoxic and non-genotoxic

Carcinogens are divided based on their mechanisms in biological systems. There are two kinds in which carcinogens are divided, and these are genotoxic carcinogens and non-genotoxic carcinogens. The difference between these two kinds is necessary in these two fields. It is important to be aware of this information in order to be able to conceptualize how cancer grows and proliferates [7]. 

Genotoxic carcinogens cause their cancer-inducing effects by directly harming the genetic material within cells. These agents generally act as electrophiles—substances that search for and attach to nucleophilic locations in DNA, resulting in the creation of DNA adducts. Such damage can lead to numerous genetic changes, such as point mutations, chromosomal abnormalities, and breaks in DNA strands. Genotoxic carcinogens are recognized as initiators of tumors because of their ability to directly alter the genome, greatly impacting the early phase of cancer development. The genetic changes they induce can activate oncogenes or inhibit tumor suppressor genes, thereby setting the stage for neoplastic transformation. Typical examples of genotoxic carcinogens are benzo[a]pyrene, aflatoxin B1, and N-nitrosamines—frequently present in tobacco smoke, tainted food, and industrial chemicals, respectively [12]. 

Certain genotoxic agents have effects that do not need metabolic activation, while others become more reactive after being transformed by liver enzymes. DNA repair mechanisms, such as those involving the protein p53 or the ATM/ATR kinase pathways, may attempt to repair the DNA. However, if the repair is ineffective or misguided, the mutations will be consolidated in the DNA and result in malignancy. Genotoxic carcinogens can be identified using conventional methods such as genotoxicity testing, including the Ames test, chromosomal aberration, and/or micronucleus tests, which examine the ability of the chemical to induce genetic mutations [7, 12–15]. 

On the other hand, non-genotoxic carcinogens (NGTX) do not target DNA directly. They do not cause mutations either. Instead, they induce cancer growth by affecting cellular processes that help cancer cells grow. They work as tumor promoters, not as initiators. Carcinogens of this nature tend to be activated metabolically by cytochrome P450. They work by affecting cellular signaling, inflammation, and hormones. They do not cause genetic mutations. Instead, they cause epigenetics, receptor responses, and issues related to intercellular communications—a phenomenon that occurs in gap junction intercellular communications (GJIC) [16]. 

Primary mechanisms of action for NGTX carcinogens include pro-mitogenesis (enhanced proliferative activity), cytotoxicity with ensuing regenerative hyperplasia, inhibition of apoptosis, oxidative stress, and endocrine dysfunction. One of the most significant mechanisms of action would be that of 2,4,4-trimethylpentane (TMP), which causes renal tumors in male rats by binding to α2u-globulin, which is found specifically in male rats, leading to chronic lysosomal distension, necrosis, and compensatory hyperplasia, ultimately progressing to tumor formation. Notably, this particular mechanism has both species-, sex-, and mechanism-specific patterns, reflecting the complexity of NGTX’s role in carcinogenic processes [17]. 

Non-genotoxic carcinogens encompass phenobarbital, which induces liver tumors by activating the constitutive androstane receptor (CAR), and TPA (12-O-tetradecanoylphorbol-13-acetate), a potent promoter of skin tumors that stimulates protein kinase C (PKC) pathways.

In contrast to GTX agents, NGTX carcinogens typically yield negative results in genotoxicity tests, resulting in their detection being more challenging with traditional screening techniques. As a result, extensive carcinogenicity studies in animal models are usually necessary to assess their potential to cause cancer [18]. 

Genotoxic and non-genotoxic carcinogens both contribute to cancer but function via different biological mechanisms. GTX carcinogens cause cancer by directly changing DNA, while NGTX carcinogens facilitate tumor formation by influencing the cellular environment and regulatory processes. Comprehending both categories is crucial for thorough risk evaluation, regulatory choices, and the creation of focused approaches for cancer prevention and treatment [19–24] (Table 1).

**Table 1:** Detailed comparative table that outlines the characteristics of genotoxic (GTX) and Non-Genotoxic (NGTX) carcinogens based on their mechanisms of action [6, 25–27]

Feature	Genotoxic Carcinogens (GTX)	Non-Genotoxic Carcinogens (NGTX)
Primary Mechanism	Directly damage DNA or chromosomes	Do not damage DNA directly; affect cellular processes
Mode of Action	Act as electrophiles forming DNA adducts	Act through biotransformation and cellular signaling
Target Molecule	DNA	Proteins, signaling molecules, cell receptors
Tumor Role	Tumor initiators	Tumor promoters
Genetic Impact	Cause mutations, chromosomal aberrations	Do not cause direct mutations
Biotransformation	Not always required	Often require metabolic activation (e.g., via cytochrome P450 enzymes)
Cellular Effects	DNA strand breaks, gene mutations, cross-linking	Altered signal transduction, increased proliferation, inflammation
Typical Outcomes	Initiation of carcinogenesis through DNA alteration	Promotion and progression of tumors through non-DNA mechanisms
Associated Pathways	DNA damage response pathways (e.g., p53, ATM)	Pro-mitogenic pathways, receptor interaction, and inflammatory response
Examples of Effects	DNA adduct formation, mutagenesis	Cell growth, division, immunosuppression, hormonal disruption
Examples of Compounds	Benzo[a]pyrene, aflatoxin B1, N-nitrosamines	Toluene-2,4-diisocyanate, phenobarbital, TPA, TMP (2,4,4-Trimethylpentane)
Key Enzymes Involved	DNA polymerases, repair enzymes	Cytochrome P450 enzymes, receptor kinases
Experimental Indicator	Positive in genotoxicity assays (e.g., Ames test)	Often negative in genotoxicity assays; require long-term bioassays
Case Example	Aflatoxin B1 forms adducts with guanine residues in DNA	TMP binds to alpha 2u-globulin in male rats, causing lysosomal overload, necrosis, and regenerative proliferation leading to renal tumors
Risk Assessment	Usually considered high risk even at low doses	Risk depends on chronic exposure and cumulative effects

## Chemotherapeutic induction of organ-specific tumors

Carcinogens can be classified into two categories: complete and incomplete, based on their roles in the various stages of cancer development.

Complete carcinogens can independently initiate cancer without the need for additional tumor-promoting agents. This means they act as both initiators and promoters when given at the appropriate doses and for sufficient durations of exposure. A well-known example of a complete carcinogen is polycyclic aromatic hydrocarbons (PAHs).

On the other hand, incomplete carcinogens are mutagenic agents that can cause irreversible DNA damage or promote the growth of benign lesions, but they cannot initiate malignant transformation on their own. These agents typically require the presence of a promoting factor to advance the progression to cancer [28, 29].

### Classifications of chemical carcinogens

According to the chemical carcinogens category, there is a whole array of chemicals that fit into this category, such as those that result from the cooking of meats, food additives, chemicals, environmental pollutants, smoking, antineoplastic drugs, naturally occurring agents, and synthetic chemicals. All of these substances have been proved to cause cancer in test animals as a result of prolonged and excessive contact.

A) N-Nitroso compounds

N-nitroso compounds (NOCs) were first discovered because of suspected carcinogenic activity. N-nitroso compounds may be present in processed foods, contaminated water, and cigarette smoke. There have been reports regarding the spontaneous occurrence of specific N-nitroso compounds like N-nitrosodiethlamine (DENA) and N-methyl-N-nitro-N-nitrosoguanidine (MNNG), because of the use of certain nitrites along with the meat products. DENA has been proved to be a potent hepatocarcinogen. They were given as a chronic intraportal treatment because of the carcinogenesis of liver cancers in rats and monkeys. It has histopathological changes very similar to the changes caused in the liver of a patient affected by liver cancer. However, the duration of this procedure is relatively long because it may take weeks [30].

MNNG is also reported as a gastric carcinogen for the study of rodents and primates. However, the part of NDMA and DMN as a hepatocarcinogen, initiator, and mutagen has been proved for liver cancer in rats. Additionally, the part of NNK and its metabolite NNAL has been significant regarding the onset of pulmonary cancer as a biomarker of exposure to tobacco. Female A/J mice are particularly sensitive for studying lung cancer, and treatment with NNK resulted in a 100% tumor incidence after 30 weeks. Hamsters exposed to NNK also developed pancreatic cancer, although with reduced organ-specificity [26]. 

NNK exposure is directly linked to G to A transitions in the KRAS oncogene (specifically at codon 12). This mutation locks the KRAS protein in a ‘permanent ON’ state, constitutively activating the MAPK/ERK signaling pathway, which drives the uncontrolled proliferation observed in lung adenocarcinoma models [27]. 

N-Butyl-N-(4-hydroxybutyl) nitrosamine (BBN) reliably causes high-grade, invasive squamous cell urinary bladder cancers that closely resemble human bladder cancer based on histopathological findings. In mice, every treated animal showed bladder cancer within 20 weeks of BBN exposure. Conversely, in rats, there was a notable onset of bladder cancers after 6–8 months of a single exposure to BBN in their potable water. In a two-step model, phenyl ethyl isothiocyanate (PEITC) has been demonstrated to enhance the formation of transitional cell carcinomas when combine with BBN administration [31–37].

B) Heterocyclic amines

Preparing red meats at elevated temperatures for longer durations may result in the formation of several cancer-related substances referred to as heterocyclic amines (HCAs). These are a set of mutagenic substances that have demonstrated an elevated cancer risk in lab animals. The HCAs are frequently examined in animal cancer models:

- 2-amino-3-methylimidazo[4,5-f]quinoline (IQ)
- 2-amino-3,8-dimethylimidazo[4,5-f]quinoxaline (MeIQx)
- 2-amino-1-methyl-6-phenylimidazo(4,5-b)pyridine (PhIP)

The major form of HCA was found to be PhIP, and it has been well known for its complete carcinogenic potential. Apart from its estrogenic promoting effect, PhIP has DNA damaging/mutagenic initiating effects. Case studies conducted on rats have shown that PhIP is known to induce cancers of the breast, colon, and prostate [1, 30]. 

On the contrary, it has been proved that MeIQx has the potential to act as a risk factor not only for hepatocellular carcinoma, but also for lung carcinoma, especially during the initiation phase of rats and mice. IQ is already proved to be a genotoxic carcinogen that leads to hepatocellular carcinoma in rats, mice, as well as primates [32, 38–41].

C) Polycyclic aromatic hydrocarbons

If grilling and roasting, the meat can create polycyclic aromatic hydrocarbons, or PAHs, which are very carcinogenic. A few examples of such chemicals are 2-acetylamino-fluorene, or 2-AAF; 7,12-dimethylbenzanthracene, or DMBA; benzanthracene, or BaA; benzopyrene, or BaP; and 3-methylcholanthrene, or MCA.

2-AAF has widely been utilized as the initiator in Solt-Farber protocol for multistage experimental liver carcinogenesis in rats. This protocol involves initiating hepatocytes along with one intraperitoneal injection of diethylnitrosamine (DENA), and then promoting them with either a diet containing 2-AAF or 4 to 6 single gavage administrations of 2-AAF. After this, the animals undergo a partial hepatectomy in order to stimulate the initiated hepatocytes to proliferate. The placental glutathione S-transferase (GST-P) is commonly used to recognize preneoplastic focal lesions in liver cancer and is a marker enzyme in estimating various chemopreventive agents. Furthermore, 2-AAF has been reported to induce liver mutations in mice [1, 12]. 

DMBA is a fully formed carcinogen that is especially potent in causing breast cancer in female Sprague-Dawley (SD) rats. A single gavage administration of 50 to 100 mg/kg of DMBA can result in mammary tumors with a 100% occurrence rate. The resulting tumors are morphologically diverse and reliant on hormones. In the squamous carcinogenesis model of mouse skin, DMBA acts as the initiator paired along with the promoter 12-O-tetradecanoylphorbol-13-acetate (TPA). In skin and mammary models, DMBA induces a specific A to T trans version at codon 61 of the HRAS gene. This genomic alteration activates the PI3K/AKT/mTOR pathway, promoting sustained cell survival and resistance to programmed cell death [13]. 

BaP, an important marker of PAHs, is recognized for causing cancers in the lungs, stomach, colon, and various other organs. Chlorinated derivatives of BaA, like Cl-BaA, have been shown to display organ-specific distribution and increased accumulation relative to their parent compounds, demonstrating carcinogenic effects in multiple organs, including the liver. MCA serves as a trigger for lung cancer in rodents, usually succeeded by the promoter butylated hydroxytoluene (BHT) [32, 38, 42–45].

D) Potassium bromate

Potassium bromate (KBrO₃) was once widely used as a flour improver and food additive in the bread production process. Additionally, bromate is a disinfection by-product formed in drinking water. In rat models of renal cancer, KBrO₃ acts as a complete carcinogen [46].

E) Polychlorinated biphenyls

Polychlorinated biphenyls (PCBs) are cancer-causing agents that are classified as dioxin-like compounds. These substances are generated as by-products in industrial processes and are frequently present in the environment as pollutants. The compound that has been studied the most in this category is 2,3,7,8-tetrachlorodibenzo-p-dioxin (TCDD), recognized for lacking direct genotoxic effects. Rather, TCDD serves as a tumor promoter across different locations and species. This effect is thought to be facilitated through two primary pathways.

Initially, TCDD can stimulate the aryl hydrocarbon receptor (AhR), resulting in the transcription of cytochrome P450 enzymes and the induction of oxidative stress. Secondly, it may interfere with apoptosis signaling pathways, including Akt and ERK, leading to a reduced rate of apoptosis [30, 47].

F) N-Methyl-N-nitrosourea

Initially designed as chemotherapeutic alkylating agent, N-Methyl-N-nitrosourea (MNU) has shown direct carcinogenic impacts on breast tissue. One administration of MNU has been demonstrated for triggering breast cancer in Sprague-Dawley (SD) rats. Although mammary cancers induced by both MNU and 7,12-Dimethylbenz[a]anthracene (DMBA) are typically hormone-dependent, those induced by MNU tend to be locally aggressive and exhibit a strong propensity for spontaneous metastasis. This trait renders them especially valuable for examining malignant progression or advanced phases of breast cancers, as they markedly differ from tumors induced by DMBA. Moreover, MNU has been demonstrated to encourage several other cancer types in experimental animals, such as esophageal, gastric, and colorectal tumors, and it can also trigger prostate and thyroid cancers [48, 49].

G) Naturally occurring compounds

I. Aflatoxins are natural mycotoxins produced by the Aspergillus flavus or Aspergillus parasiticus species. These are very common in foods, and there is contamination of foods as an environmental problem all over the world. Aflatoxin B1 (AFB1) has been found to be highly lethal to the liver, displaying potent mutagenicity [50, 51]. AFB1 serves as a classic example of a carcinogen-specific mutation; it specifically induces a G to T transversion at codon 249 of the TP53 tumor suppressor gene. This ‘signature mutation’ disrupts the p53 pathway, leading to a critical loss of cell cycle control and apoptosis in liver cells [14].
II. The kind of asbestos that has been extensively used in industry is composed of different kinds of fibers that occur in soil and rock. These have been observed to produce oxygen-derived radicals that contribute to DNA double-strand breaks and toxicity in cells. Asbestos has been given in murine models of pleural or peritoneal malignancies of mesothelioma in experiments in which intraperitoneal or intra-tracheal instillation has been performed [52, 53].
III. Aristolochic acids (AAs) were identified as key compounds extracted from the Aristolochia plant, which was used in the old herbal remedy but was later identified as potent nephrotoxins by Belgians in 1991. AA is a potent carcinogen in rodents, and long-term oral administration of AA in rats and mice has been shown to lead to multiple tumors, primarily in the forestomach and kidney [54, 55].

H) Synthetic carcinogens

(I) DMH, AOM, and MAM are synthesized molecules that have cycasin-like structure, which demonstrate complete carcinogenic activity in animal models of colon cancer [56].
(II) 4NQO, a quinolone derivative, is a complete carcinogen that has been widely used in research studies. It has the ability to cause all stages of oral squamous cell carcinoma that are very similar to human cancers [57].
(III) ENU, an extremely potent mutagen, was used as a neurotropic carcinogen to establish the glioma rat model and to enhance the incidence and multiplicity of mammary tumors in mice with a mutant Apc tumor suppressor gene as well as to induce hepatic mutation in infant Big Blue transgenic mice [58].
(IV) BHP, a hydroxylated dipropylnitrosamine, displays complete carcinogenicity by mechanisms varying with route of administration and species tested. It is pancreatic carcinogenic in Syrian golden hamsters given subcutaneously, whilst it is pulmonary carcinogenic in Wistar rats dosed orally [59].
(V) Phenobarbital (PB) is a synthetic non-genotoxic carcinogen/promoter and is often used to promote the carcinogenesis of the liver and kidneys [25].

## Experimental applications

The chemicals listed account for merely a small portion of the numerous identified and undiscovered carcinogens. Their experimental application remains in the initial, primarily empirical phases, offering considerable avenues for additional investigation. Nonetheless, primary cancer models remain essential instruments for various fundamental and translational research efforts. These models aid in comprehending the biological and molecular processes that play a role in tumor initiation, promotion, and advancement. They additionally assist in recognizing impactful and protective elements in cancer progression and in assessing the diagnostic and treatment capabilities of potential drugs utilizing induced primary tumor models [25].

### Carcinogenic process involves related genes and pathway

The utility of chemically induced models lies in their ability to replicate specific human mutational landscapes. Rather than generic damage, different carcinogens target distinct genes and signaling cascades. For example, colon cancer models induced by Azoxymethane (AOM) specifically target codons 32–34 of the CTNNB1 (Beta-catenin) gene. This alteration prevents the degradation of beta-catenin, causing its nuclear accumulation and the subsequent over-activation of the Wnt/β-catenin signaling pathway, mimicking human sporadic colorectal cancer. Scientists employ various multistage carcinogens to examine the molecular alterations linked to the initiation and promotion stages of cancer progression [13–17]. 

Recent research has discovered numerous genomic changes and somatic mutations in oncogenes and tumor suppressor genes in diverse human cancers. Nonetheless, the specific elements that cause these mutations remain largely unclear. For instance, alterations in the epidermal growth factor receptor (EGFR) gene are frequently found in human lung adenomas and adenocarcinomas. Rodent models of lung cancer, caused by substances such as NNK, BHP, MeIQx, urethane, and X-ray, are useful for studying mutation trends in the Egfr and K-ras genes. These patterns can be matched with related human lesions to enhance the understanding of the regulatory mechanisms at play [13–17]. 

In the case under evaluation, genetic mutations can be utilized as bio-markers that have a strong molecular basis. The expression of the placental glutathione s-transferase protein in the liver models has been viewed as a bio-marker for the preneoplastic stage, utilized to determine the stage of preneoplastic change prior to the occurrence of the outbreak. The application of bio-markers in the preclinical stage allows for the selection of the high-risk stage, indicating the start of the carcinogenesis process in human neoplastic disease [17]. 

In addition to that, there are also comparative studies that can be done to establish the genes and their expressions that are impacted by the development of cancer, as well as new targets in the treatment of the disease. Also, the use of genetically modified mice with the application of genes such as p16, p19, p53, Apc, c-Ha-ras, myr-Akt, TGF-alpha, and Klf6 provides an innovative way to research, especially in the event that the mice are exposed to carcinogens. In the mice models, the researchers are able to accurately identify the specific role played by the genes implicated in the development of cancer, as well as important leads in the modulation of signal pathways to cancer [25, 32]. Table 2 Specific Carcinogen-Induced Gene alterations and Signaling Pathways.

**Table 2:** Specific Carcinogen-Induced gene alterations and signaling pathways

Carcinogen	Major Organ Target	Primary Gene Alteration	Associated Signaling Pathway	References
Aflatoxin B1	Liver	*TP53* (Codon 249 transversion)	p53-mediated Apoptosis	[26, 27]
NNK (Tobacco)	Lung	*KRAS* (G→A transition)	MAPK/ERK Signaling	[26, 27]
DMBA	Skin/Breast	*HRAS* (A→T at Codon 61)	PI3K/AKT/mTOR	[26, 27]
AOM	Colon	*CTNNB1* (Beta-catenin)	Wnt/β-catenin Pathway	[26, 27]
DENA	Liver	*Hras* and *Myc* amplification	Ras/Raf/MEK	[26, 27]

### Cancer preventive and influential factors for tumorigenesis

Despite the breakthroughs in the treatment of cancer, the rate of incidence and mortality caused by cancer continues to escalate around the world, as reported by the World Health Organization. There comes the need to accelerate the research efforts in the prevention of cancer. In the prevention of cancer research trials, among the approaches that show great promise is by the implementation of chemoprevention, which involves the use of specific chemicals to inhibit, prevent, or even reverse the onset of cancer in its early stages through the influence of the carcinogenic process. In the use of the agent in the prevention or conduction with the carcinogen in the animal model, it would be possible to establish its efficiency [7, 13]. 

Additionally, in recent years, the use of natural compounds from medical plants has received much attention regarding their potential ability to function as chemopreventive agents, mainly because of their high antioxidant and anti-inflammatory activities, largely responsible for the attenuation of the toxic potential of carcinogenesis-induced oxidative stress. For instance, lutein, the green carotenoid pigment of green leafy vegetables, could inhibit hepatic preneoplastic alteration during the promotion and DNA damage phase of chemically induced rat hepatocarcinogenesis. Similarly, compounds such as resveratrol, curcumin, capsaicin, and allicin stated as nutraceuticals currently under extensive clinical trial investigation that has demonstrated effectiveness in countering cancer initiation and progression [17, 18]. 

Other naturally occurring substances have also shown potential in cancer prevention. For example, Astragalus membranaceus, a traditional Chinese medicinal herb used to treat liver diseases, and betaine, a compound found in wheat, spinach, and sugar beets, have been reported to delay hepatocarcinogenesis in rodent models. Additionally, the aqueous extract of dried Anoda compressa leaves—commonly used as a traditional herbal tea in Mexico—has exhibited notable chemo preventive effects [32, 34, 42].

### Bioinformatics and AI in carcinogenesis research

Modern risk assessment is increasingly reliant on AI-assisted methodologies to bridge the gap between animal data and human clinical outcomes. AI algorithms are now used to distinguish between genotoxic and non-genotoxic carcinogens by analyzing chemical structures through QSAR modeling and toxicogenomic data. Deep learning models, such as convolutional neural networks (CNNs), are being used in 2025 to automate the analysis of comet assays and micronucleus tests, significantly reducing the 40–60% false-positive rates seen in manual mammalian cell assays [17, 18]. 

Furthermore, bioinformatics platforms allow researchers to compare mutations found in mammalian models with human clinical data. Multi-omics integration (transcriptomics, proteomics, and metabolomics) assisted by machine learning helps identify ‘core targets’ and signaling pathways. For instance, recent 2025 studies have used AI to identify specific microRNA signatures (like miR let-7) that drive tumor transformation in non-genotoxic models as mentioned in Table 3.

**Table 3:** AI and bioinformatics tools in chemical carcinogenesis

Category	Tool/Method	Clinical/Research Application	References
Toxicity	ProTox 3.0/ADMETlab 3.0	Predicting carcinogenicity using SMILES structures	[1]
Early Detection	CNN-based Imaging	Automated detection of metastasis and small lesions in MRI.	[2]
Genomic Analysis	HNNMixCancer	Hybrid Neural Networks to assess risk of chemical mixtures.	[3]
Drug Repurposing	PLpro Inhibitor Screening	Combining ML and molecular dynamics for new targets.	[60]

## Preclinical evaluations of new specific contrast agents

Early diagnosis and accurate staging of various cancers are critical for initiating timely clinical interventions and appropriate palliative therapies. Diagnostic imaging plays an essential role in providing detailed anatomical and functional information to facilitate these objectives. However, one of the major limitations in current imaging techniques is the relatively poor soft-tissue contrast between malignant lesions and adjacent normal tissues. To address this challenge, recent preclinical research has focused on the development of novel, tissue-specific contrast agents, which have expanded the utility of imaging modalities beyond structural visualization to include functional and molecular imaging [25].

Of these modalities, magnetic resonance imaging (MRI) has emerged as the premier diagnostic technique owing to its high soft tissue contrast without the need for invasive procedures, which makes it an important tool in the diagnosis of cancers. Additionally, the use of liver-specific contrast agents, including Manganese Dipyridoxyl Diphosphate (Mn-DPDP) and Gadoxetic acid (Gd-EOB-DTPA) which are the current established liver-specific MR imaging agents, has shown effectiveness in the diagnosis of multifocal liver tumors in DENA-induced rat models of liver cancer. Compared to conventional agents Gd-DTPA, Gd-DOTA, these liver-specific agents have shown greatly improved image quality that helps in precise visualization of tumors [26, 30].

Beyond anatomical imaging, the use of tissue-specific contrast agents like Mn-DPDP and Gd-EOB-DTPA effectively turns MRI into a tool for functional biomarker assessment. These agents act as biomarkers by highlighting early metabolic and structural alterations in the liver, enabling the early detection of multifocal tumors that might otherwise be missed by conventional imaging [1]. 

The integration of targeted contrast agents in MRI represents a promising advancement in cancer diagnostics. By providing improved lesion detectability and anatomical clarity, these agents facilitate more accurate tumor detection, staging, and treatment planning. Continued research in this field is expected to yield next-generation contrast agents with enhanced specificity and sensitivity, ultimately improving diagnostic accuracy and therapeutic outcomes in oncology [30].

Beyond hardware and contrast agents, AI-driven image analysis is revolutionizing the use of MRI in small animal models. Algorithms for automated segmentation and quantitative feature extraction (radiomics) allow for the detection of tumors with an accuracy that matches or exceeds human radiologists. This AI integration enables earlier identification of preneoplastic lesions and more precise longitudinal monitoring of therapeutic responses in primary cancer models [7, 12, 13]. 

### Therapeutic strategies for primary malignancies

Chemically induced cancer models have become essential tools for evaluating the effectiveness of various therapeutic interventions in both preclinical studies and fundamental cancer research. These models are particularly beneficial because they more accurately replicate the multistep process of carcinogenesis seen in clinical settings, encompassing the initiation, promotion, and progression phases.

A notable example is a study that explored the therapeutic potential of Rapamycin using a two-stage mouse skin tumor model. Rapamycin (Sirolimus) and its analogs are FDA-approved mTOR inhibitors, and their successful testing in two-stage skin models directly reflects their clinical utility in treating various human malignancies [61]. In this model, tumor initiation was achieved with a single application of 7,12-dimethylbenzanthracene (DMBA), followed by repeated administration of phorbol esters to promote tumor development. This setup allowed researchers to assess the therapeutic effects of Rapamycin at both early (9–10 weeks) and advanced (approximately 16–18 weeks) stages of carcinogenesis. The findings indicated that chemically induced models provide a more realistic and dynamic platform for analyzing drug effects over time and throughout different phases of tumor progression [30]. To further illustrate the translational utility of these models, Table 4 summarizes key therapeutic and diagnostic agents—ranging from those in late-stage clinical trials to FDA-approved treatments—that have been validated or developed using similar mammalian systems.

**Table 4:** Clinical and trial agents in chemical models [62, 63, 64, 65, 66]

S. No.	Drug Name	Clinical Phase/Status	Target Indication
1	Vimseltinib	Approved (Feb 2025)	Tenosynovial giant cell tumor
2	Vepdegestrant (ARV-471)	Phase 3 (VERITAC − 2)	ER+/HER2-Breast Cancer
3	Darovasertib	Phase-3 (OptimUM-10)	Uveal Melanoma
4	Relacorilant	Phase 3 (ROSELLA)	Ovarian Cancer
5	Zongertinib	Approved (Sept 2025)	HER2-mutated NSCLC
6	Imlunestrant	Approved (Sept 2025)	ESR1-mutated Breast Cancer
7	Amezalpat (TPST-1120)	Phase 2 (Fast Track)	Liver Cancer (HCC)`
8	Revumenib	Approved (Oct 2025)	NPM1-mutated Leukemia
9	Dordaviprone	Approved (Aug 2025)	H3 K27M-mutated Glioma
10	Linvoseltamab	Approved (Aug 2025)	Multiple Myeloma
11	Zolbetuximab	Phase 3	Gastric/GEJ Adenocarcinoma
12	Pelabresib	Phase 3	Myelofibrosis
13	BNT142 (mRNA-based)	Phase 1/2	Testicular & Ovarian Cancer
14	VLS-1488	Phase 1/2	KIF18A-mutated Solid Tumors
15	CUSP06 (ADC)	Phase 1 (Fast Track)	Ovarian Cancer
16	AUTX-703	Phase 1 (Starts 2025)	Acute Myeloid Leukemia
17	PYX-201 (ADC)	Phase 1/2	Head and Neck Cancer
18	Pasritamig	Phase 2	Prostate Cancer
19	Abenacianine	Phase 2 (Fast Track)	Lung Cancer (Imaging/Surgery)
20	Sevabertinib	Approved (Nov 2025)	Non-Squamous NSCLC
21	Tarlatamab	Traditional Approval	Small Cell Lung Cancer
22	Bepirovirsen	Phase 3 (B-Well)	Chronic Hep B (Liver Cancer)
23	Selinexor	Phase 3 (Maintenance)	Endometrial Carcinoma
24	Inotuzumab Ozogamicin	Phase 3	High-Risk B-ALL
25	AZD1390	Phase 1	Pediatric Brain Tumors
26	FPI-2265	Phase 2/3	Castration-Resistant Prostate

### Advantages and disadvantages

Chemically induced primary malignancies in mammalian models are widely employed to simulate human cancers due to their simplicity, cost-effectiveness, and high tumor induction rates. These models are capable of generating a broad spectrum of primary tumors that vary in size, degree of differentiation, and even tumor origin. A representative example is diethylnitrosamine (DENA)-initiated hepatocarcinogenesis in rodents, which leads to the formation of multifocal primary liver tumors against a background of liver cirrhosis. This allows for comparative analysis of benign versus malignant lesions, tumor tissue versus adjacent non-tumorous regions, thereby enhancing the evaluation of therapeutic agents and supporting the development of novel diagnostic imaging techniques [7, 60]. 

These models mirror the clinical course and pathological progression of various human cancers, with similarities in morphology, histopathological features, and underlying molecular alterations. Furthermore, different carcinogens can induce distinct subtypes of cancer with varying biological characteristics. For example, 7,12-dimethylbenz[a] anthracene (DMBA) and N-Methyl-N-nitrosourea (MNU) are commonly used to induce mammary tumors. While DMBA-induced tumors are largely hormone-dependent, those induced by MNU are often hormone-independent and more aggressive, making them useful for studying advanced cancer stages [17]. 

However, these models also have certain limitations. One major drawback is the unpredictable and heterogeneous nature of tumor development, in terms of timing, anatomical location, tumor size, histological differentiation, and vascularization. This presents significant challenges in the non-invasive monitoring of tumor burden, especially in visceral organs such as the liver, lungs, kidneys, and pancreas. As a result, it becomes difficult to determine accurate grouping, treatment schedules, and study endpoints. In many experimental protocols, particularly those evaluating carcinogenesis, chemoprevention, or therapeutic efficacy, final tumor assessment often relies on histopathological examination following the sacrifice of animals or, in some cases, interim biopsies to gather supplementary data [25, 30].

## Future direction and clinical outlook

I. Artificial Intelligence and Radiomics: “The future of clinical oncology is to be found in the field of ‘Radiomics’-a process where massive amounts of data are gleaned from MRI via AI. Future research will be well spent in training machine learning algorithms upon the mammalian models discussed herein to predict tumor aggressiveness and genetic mutations, such as KRAS or TP53, in a noninvasive manner before biopsy [7, 12–15].
II. Precision Prevention and Liquid Biopsies: “While the current state of the art is aimed at late-stage disease, these models represent the roadmap to the reality of ‘Precision Prevention.’ Using these models to zero in on early stage Volatile Organic Compounds or Circulating Tumor DNA during the promotion phase, we could create ‘liquid biopsies’ for the 2026 applications of our clinical model to detect cancer at the earliest, most treatable stage [16].
III. Development of ‘Smart’ Contrast Agents: “In the clinics, we predict a shift from conventional anatomical contrast agents to ‘smart’ molecular contrast agents for MRI imaging applications,” said guest contributor. “This contrast agent would not only indicate the location of cancer, but also would allow one to know the values of pH, oxygen level, or activity of an enzyme like GST-P in the cancer in real time, to enable an adjustment of chemotherapy accordingly [17].
IV. The Species Gap: Multi-Omics Bridging. “A significant future direction is the use of bioinformatics to create a ‘translational bridge’ between the murine and human signatures. Through the use of cross-referencing between the particular gene-pharmaceutical interactions as outlined within table Y, to human data banks like the TCGA, one can ensure that those pharmaceutical agents validated through the model possess a significant ‘success’ factor’ during the phase I/II human clinical trials [18].

## Conclusion

Chemically induced primary cancer models in mammals stand as indispensable tools for understanding carcinogenesis mechanisms and evaluating novel diagnostic, preventive, and therapeutic strategies in preclinical settings. Their main strength lies in faithfully recapitulating the stages of human cancer development—from initiation through promotion to progression—offering vital insights into molecular drivers of tumorigenesis and the interplay of genetic and environmental factors.

These models deliver several distinct advantages. They are simple and cost-effective to implement, allowing carcinogen administration by various routes (injection, gavage, dietary supplement, etc.) without specialized facilities or labor-intensive techniques. Chemically induced cancer models can generate a high tumor burden and produce multifocal lesions with histopathological complexity, mirroring the heterogeneity observed in clinical cancer cases. This enables simultaneous study of benign and malignant transformation, supports rigorous evaluation of drug candidates, and facilitates direct comparison between tumor tissues and healthy counterparts in the same animal.

Moreover, the diversity of carcinogens available including genotoxic and non-genotoxic agents, food contaminants, pollutants, and synthetic compounds permits the modeling of a wide spectrum of organ-specific, molecularly distinct cancer types. These models enable exploration of preventative measures, influential biological factors, and the impact of specific genomic changes on cancer development. The utilization of advanced imaging, particularly MRI, has helped overcome the major bottleneck of noninvasive tumor detection and monitoring, providing superior soft-tissue contrast, multi-parametric data, and effective use of contrast agents for staging and therapeutic evaluation. Furthermore, these models unlock opportunities for screening and validating new early-detection biomarkers. By identifying enzymatic and genomic changes during the reversible promotion phase, researchers can develop diagnostic strategies that target cancer at its most treatable stage.

Despite these strengths, the models face notable challenges: the unpredictable nature of tumor development regarding anatomical locations, timing, and multiplicity the time-consuming induction period and difficulties in noninvasive assessment, especially in smaller animals. These limitations complicate group allocation, endpoint determination, and longitudinal monitoring.

In summary, chemically induced mammalian cancer models, particularly when integrated with cutting-edge imaging and molecular profiling, remain essential for bridging basic cancer biology and clinical translation. They unlock opportunities not only for dissecting the pathophysiology of neoplasia but also for screening and validating new diagnostic agents and therapies, and for identifying strategies to prevent carcinogenesis. Continued refinement including better imaging modalities and humane study designs will further enhance their translational impact and help address outstanding gaps in cancer research.

## Data Availability

No datasets were generated or analysed during the current study.

## Abbreviations

AI: Artificial Intelligence
AOM: Azoxymethane
AFB1: Aflatoxin B1
CNN: Convolutional Neural Network
ctDNA: Circulating Tumor DNA
CTNNB1: Catenin Beta 1
DENA: Diethylnitrosamine
DMBA: 7,12-Dimethylbenz[a]anthracene
Gd-EOB-DTPA: Gadoxetic acid (Gadolinium-ethoxybenzyl-diethylenetriamine pentaacetic acid)
GEMs: Genetically Engineered Models
GST-P: Placental Glutathione S-transferase
Mn-DPDP: Mangafodipir trisodium
MRI: Magnetic Resonance Imaging
NAMs: New Approach Methodologies
NGCs: Non-genotoxic Carcinogens
NNK: 4-(methylnitrosamino)-1-(3-pyridyl)-1-butanone
NNAL: 4-(methylnitrosamino)-1-(3-pyridyl)-1-butanol
PAHs: Polycyclic Aromatic Hydrocarbons
QSAR: Quantitative Structure-Activity Relationship
TCGA: The Cancer Genome Atlas
VOCs: Volatile Organic Compounds
